# ^125^I seeds irradiation inhibits tumor growth and induces apoptosis by Ki-67, P21, survivin, livin and caspase-9 expression in lung carcinoma xenografts

**DOI:** 10.1186/s13014-020-01682-5

**Published:** 2020-10-15

**Authors:** Qing Jin, Cunzhi Lin, Xinhong Zhu, Yiwei Cao, Caihong Guo, Lijun Wang

**Affiliations:** 1 Department of Respiratory and Critical Care Medicine, The Affiliated Hospital of Qingdao University, Qingdao, 266003 Shandong Province China; 2 Department of Critical Care Medicine, The 903th Hospital of PLA Joint Logistics Support Force, Zhejiang Province, Hangzhou, 310013 China; 3 Department of Internal Medicine, Qingdao Municipal Hospital, Qingdao, 266071 Shandong Province China

**Keywords:** 125I seeds, Lung carcinoma, Apoptosis, Ki-67, P21

## Abstract

**Background:**

Lung cancer is a fatal disease and a serious health problem worldwide. Patients are usually diagnosed at an advanced stage, and the effectiveness of chemotherapy for such patients is very limited. Iodine 125 seed (^125^I) irradiation can be used as an important adjuvant treatment for lung carcinoma. The purpose of this study was to examine the role of irradiation by 125I seeds in human lung cancer xenograft model and to determine the underlying mechanisms involved, with a focus on apoptosis.

**Methods:**

40 mice with A549 lung adenocarcinoma xenografts were randomly divided into 4 groups: control group (n = 10), sham seed (0 mCi) implant group (n = 10), 125I seed (0.6 mCi) implant group (n = 10) and 125I seed (0.8 mCi) implant group (n = 10), respectively. The body weight and tumor volume, were recorded every 4 days until the end of the study. Apoptotic cells were checked by terminal deoxynucleotidyl transferase dUTP nick end labeling (TUNEL) assay and activities of caspase-3 and caspase-8 enzyme were tested. Expression of P21, survivin, livin, caspase-9 and proliferating cell nuclear antigen (Ki-67) was detected with immunohistochemical staining.

**Results:**

The results of TUNEL staining assays showed that 125I seed irradiation suppresses the growth of lung cancer xenografts in nude mice and induced apoptosis. The activity of caspase-3 and caspase-8 was significantly higher. The expression levels Ki67, survivin and livin were substantially downregulated, while P21 and caspase-9 protein expression were significantly increased following 125I seed irradiation. This study revealed that 125I seed irradiation could significantly change apoptosis-related protein in human lung cancer xenografts.

**Conclusions:**

Overall, our study demonstrates that radiation exposure by 125I seeds could be a new treatment option for lung cancer.

## Background

Primary lung cancer is the most common malignancies [1] and the leading cause of tumor-associated mortality [2–8] in gender-independent populations. Approximately 1.8 million new cases are diagnosed and near 1.6 million fatal cases are estimated annually worldwide, accounting for 19.4% of total cancer mortality, and the 5-year overall survival rate is less than 20% [2, 5, 7, 9–12] Non-small cell lung cancer (NSCLC) accounts for approximately 85% of lung cancers and small cell lung cancer (SCLC) was about 15% [3, 4, 6, 13–15], and more than 50% NSCLC are adenocarcinoma [2, 7].Surgery remains the main curative selection for patients with early-stage non-small cell lung cancer. However, less than 20% of patients with non-small cell lung cancer who have not yet been diagnosed with advanced disease can be cured by surgical resection [16]. Chemotherapy and radiotherapy (RT) are commonly used for patients who are not considered candidates for surgery. However, these modalities are not usually curative and are almost always accompanied by various toxic complications (myelosuppression, nausea, vomiting and radiation pneumonitis), especially affecting important organs and tissues (heart, esophagus, and large blood vessels) [17, 18]. Therefore, it is necessary to effectively prolong the survival time and significantly improve the quality of life in advanced patients.

125I brachytherapy has been accepted as a minimally and useful invasive treatment for different tumors with significant efficacy. Compared with conventional external radiotherapy, 125I brachytherapy has the characteristics of minimal complications, minimal invasive, high dosage in the diseased area, and less exposure to normal tissue [19–21]. It further improves the anti-tumor effect via killing tumor cells and most effectively protects normal tissue [21, 22], and therefore it has been rapidly applied in clinic practice. The most common application of 125I seeds irradiation has been in the local treatment of advanced and inoperable prostate cancer [23, 24], although therapy for other cancers, such as lung cancer, hepatocellular carcinoma, gastric carcinoma, colorectal cancer, pancreatic adenocarcinoma and head/neck cancer [19–21, 25–33].

Although many clinical trials have reported that 125I seed radiation is a feasible adjuvant treatment for controlling local symptoms of advanced or inoperable NSCLC and prolonging survival [28, 33], its molecular mechanism underlying and biological effects are far from fully understood.

## Materials and methods

### Cell culture

The human lung adenocarcinoma cell line A549 was purchased from the American Type Culture Collection (ATCC, Manassas, VA, USA). The cells were cultured in RPMI-1640 (Hyclone, Logan, UT) medium supplemented with 10% Fetal Bovine Serum (FBS) (Gibco, Carlsbad, CA, USA) and 1% penicillin–streptomycin (Hyclone) in a 37 °C incubator with 5% CO_2_. All procedures were carried out using cells were seeded at 70–80% confluence. The cells were more than 95% viable as assessed by trypan blue exclusion.

### Animal model

Female BALB/c nude mice, weighing 17–20 g and 4–6 weeks old, were purchased from Institute of Chinese Academy of Medical Sciences. Before any intervention was initiated, the nude mice were maintained in pathogen-free conditions (55 ± 5% humidity and 23 ± 2 °C) for 1 week. The study was approved by Animal Ethics Committee of Qingdao University. Nude mice were injected with 5 × 10^6^ A549 cells. Tumor size, volume and weight of the mice were calculated daily until the remainder of the experiment. We calculated the tumor volume (V) using the formula: V (mm^3^) = L × W^2^/2(W, width of tumor; L, length of tumor).

### 125I brachytherapy seeds implant

The ^125^I seeds (4.5 mm long, 0.8 mm diameter) were obtained from Qingdao University Hospital. The energy of ^125^I was an average from 27.4 to 35.5 keV, and its half-life is about 59.6 days. The ^125^I is continuously-releasing soft X-ray and low-dose-rate γ-irradiation after decaying into the organs. It is a considerably long of internal radiation, and the brachytherapy dose (93–97%) is depleted in 8–10 months. Once the tumors had reached 300 mm^3^ in size (about 24 days), mice were randomly divided into 4 groups (n = 10/group): sham seed implant group; 125I seed (0.6 mCi) implant group; 125I seed (0.8 mCi) implant group and non-implanted control group. Before cell inoculation, BALB/c nude mice were anesthetized with diethyl ether. The seeds in the form of 18-gauge needles (called Mick-applicator) were directly implanted into the visible tumor of mice. Mice were killed, and tumors from each group were collected and weighed, then fixed with 4% paraformaldehyde (PFA) after 32 days of treatment.

### Hematoxylin and eosin (H&E) staining

Tumor tissues were fixed in 4% paraformaldehyde for 24 h. After paraffin imbedding, the tissues were sliced into 4 μm-thick sections. The sections were dehydrated with gradient ethanol, and then stained with hematoxylin for 5 min. After differentiated in 1% hydrochloric acid alcohol for 2 s, the sections were then incubated in ammonia water, followed by the staining with eosin. Ultimately, the sections were dehydrated, cleared, mounted with neutral resin, and observed under light microscopy (Olympus, Japan).

### TUNEL staining

Tumor specimens were subjected to a TUNEL assay using the In Situ Cell Death Detection kit (Roche, Basel, Switzerland), according to manufacturer's instructions for detecting apoptosis. As noted above, the fixed tissues were incubated with 100 μl Proteinase K for 30 min at 37 °C. Slides were rinsed twice with PBS. Fifty microliter TUNEL reaction mixture was added to the sample in a humid and dark atmosphere at 37 °C and incubated for 60 min. Then fifty microliter Converter-POD solution was added to the sample at 37 °C incubated for 30 min. DAB substrate was added to the slides, overlaid with a coverslip and analyzed under light microscope.

The numbers of overall tumor and TUNEL positive cells were quantified in five random sections by a light microscope at magnification of 400 ×. The apoptotic index was determined as the percentage of TUNEL positive cells to overall tumor cells. Slides with DAB-stained were analyzed by an Olympus BX51TPHD-J11 microscope (Tokyo, Japan). The analysis software (Image Pro Plus, Media Cybernetics, USA) was used for image and data acquisition and analysis.

### Caspase-3 and caspase-8 activity test

According to the manufacturer’s a protocol, the caspase-3 and caspase-8 detection kits (Beyotime, Shanghai, China) were used to measure the activity of caspase-3 and caspase-8. The treated cell lysates were incubated with lysis buffer on ice for 15 min. Then they were centrifuged (13,000×*g*, 4 °C, 5 min) and the supernatants were transferred to 96-well plates. Reaction buffer, containing 10 mM DTT was added to each well and the 10 µl Ac-DEVD-*p*NA (2 mM) substrate was mixed. The mixture was incubated for 2 h at 37 °C, and then protease activity was detected using a fluorescence microplate reader at 450 nm.

### Immunohistochemistry for P21, survivin livin and caspase-9

Expression of P21, survivin, livin and caspase-9 was detected by Immunohistochemistry. Three sections were tanked from each xenograft tumors. The main procedures are as follows: after conventional deparaffinization, rehydration, and blocking of endogenous peroxidase activity for 15 min, sections were pretreated for the purpose of antigen retrieval by microwaving, and then washed with PBS. Sections were incubated for 2 h at room temperature with mouse anti-human P21 monoclonal antibody (Zsbio, Beijing, China), rabbit anti-human Caspase-9 monoclonal antibody at a 1:50 dilution (Bioss, Beijing, China), rabbit anti-human Survivin monoclonal antibody (Zsbio, Beijing, China) Company) and rabbit anti-human Livin monoclonal antibody at a 1:50 dilution (Bioss, Beijing, China) respectively. Sections were then washed three times in PBS and incubated for 15 min at room temperature with ready-to-use secondary biotinylated antibodies PV-9000. After that, sections were rinsed with PBS, developed with DAB, counterstained with hematoxylin, cleared with xylene and observed under a light microscope. A negative control was designed by using PBS instead of primary antibody and a known positive section was served as a positive control. All above mentioned procedures were performed in the same conditions.

Two investigators evaluated the IHC-stained tissue sections and photographed representative regions with Kawasaki et al. [34] using a microscope. A mean percentage of positive tumor cells was determined in at least five areas at × 400 and assigned to one of five of categories: (a) 0. < 5%; (b) 1. 5–25%; (c) 2. 25–50%; (d) 3. 50–75%; and (e) 4. > 75%. According to cell staining intensity score: (a) cell no color, 0 points; (b) straw colored, 1 point; (c) brownish-yellow, 2 points; (d) tan, 3 points. According to these indicators divided into four, that is negative for the 0–1 points, weak positive 2–3 points, positive 4–5 points, strong positive 6–7 points.

### Statistical analysis

All experiments were performed in three parallels and repeated at least thrice. All data were conducted using SPSS 22.0 software (IBM, Cary, NC, USA). Continuous variables were examined for normality. Results were presented as mean ± standard deviation (SD). Data did pass tests of equal variance. Multiple group comparisons were evaluated by one-way ANOVA. Comparison of means were made by Student–Newman–Keuls test. Differences in proportions were evaluated by chi-square test. Correlations were analyzed by the Spearman rank-correlation coefficient. Difference with *P* < 0.05 were considered statistically significant.

## Results

### Effect of 125I seed irradiation on tumor growth of lung cancer

We evaluated the antitumor effects of the 125I seeds by A549 cells tumor xenografts of human lung adenocarcinoma. Tumor xenografts were established subcutaneously in 40 nude mice. When the tumors reached a mean volume of 250–350 mm^3^ without ischaemic necrosis after 3 weeks, we initiated experiments. After 30 days, the mice were euthanized and tumors were analyzed and all mice survived without cachexia and severely radiation injury. There was no obvious hemorrhagic necrosis and fibrosis of heart, lungs, spleen, liver, and kidneys. All particles were located near the middle of the tumor xenografts, and successfully recovered.

**Fig. 1:**
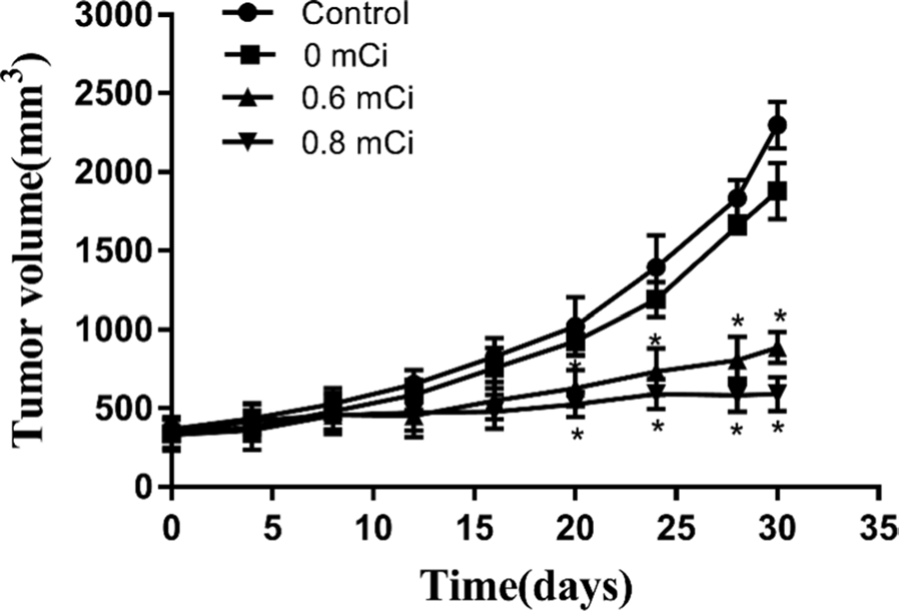
Mean tumor volumes of control and seed implant tumors on day 30 after seed implantation. Compared with the control group, *P < 0.05

To confirm the tumor suppression of 125I, we tested tumor growth (Fig. 1). In the case of xenograft tumors, median volume was 886 mm^3^ ± 97 in the in the 0.6 mCi group and 590 mm^3^ ± 107 in the 0.8 mCi group, which was different (*P* < 0.001) compared with the control group (2297 mm^3^ ± 149).There was no significant difference between 0 mCi (1779 mm^3^ ± 276) and control group (*P* > 0.05). The 0.6 mCi groups were no different from 0.8 mCi group (*P* > 0.05).

**Fig. 2:**
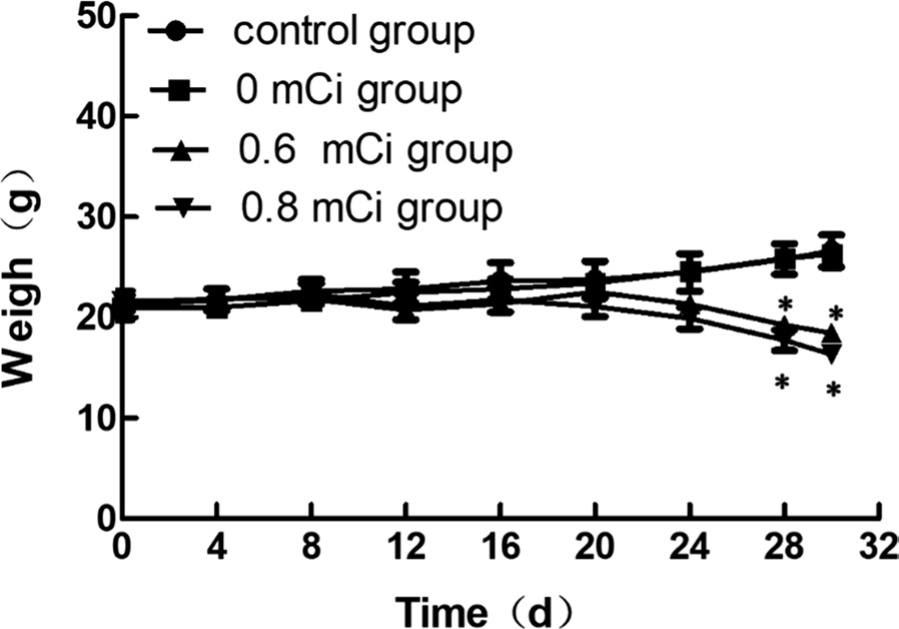
Mean tumor weight of control and seed implant tumors on day 30 after seed implantation. Compared with the control group, *P < 0.05

The weight of nude mice remained stable after particle implantation in all groups. However, the weight decreased in the mice of 0.6 and 0.8 mCi groups over time, and there was no difference compared with the control group (*P* > 0.05). The weight of nude mice in the 0.6 and 0.8 mCi groups was less than that in the control group on the 30th day (*P* < 0.05), but there was no significant difference between the 0.6 and the 0.8 mCi group, 0 mCi and the control group (*P* > 0.05) (Fig. 2).

The tumor weights in the 0.6 mCi (1.20 ± 0.44)g and 0.8 mCi (0.99 ± 0.404)g groups were less than the control group (2.35 g ± 0.64, *P* < 0.05 for all comparisons).

A comparison between 0.8 and 0.6 mCi group was no significant (*P* > 0.05). There was no statistically significant difference between 0 mCi (2.26 ± 0.53)g and the control group (*P* > 0.05). The growth inhibition of tumor rates was 49% in 0.6 mCi group and 62% in 0.8 mCi group (Fig. 2).

### Histopathological alterations in xenograft tumors

**Fig. 3:**
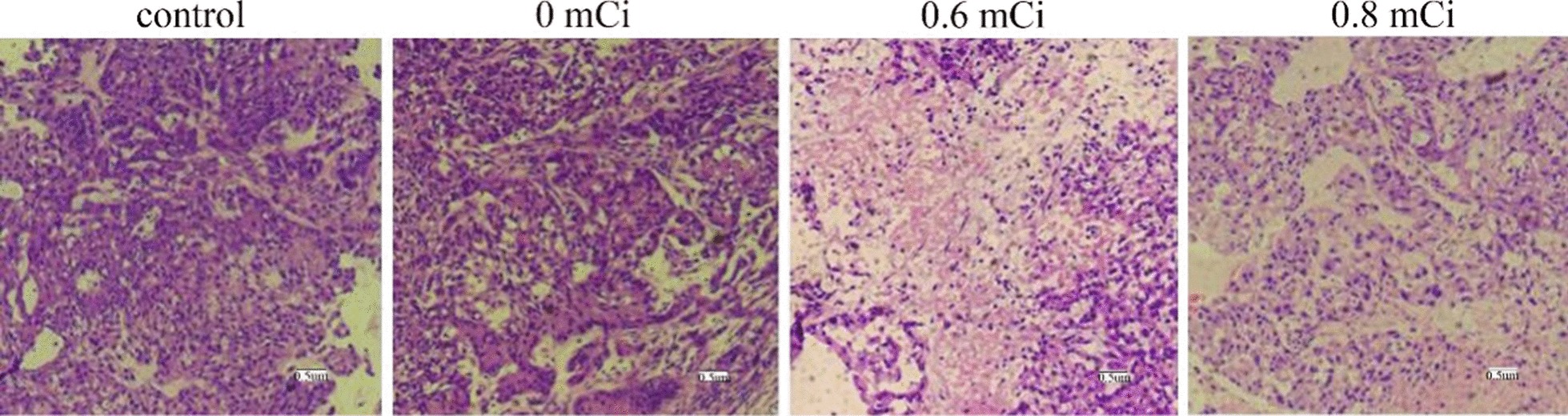
Sections of tumors developing in nude mice stained with HE (× 100)

Hematoxylin and eosin (H&E) stained sections showed the tumor cells as red, the blood vessels and the stroma blue. The H&E stained sections were observed abundant tumor cells and stroma. The tumor cells with closely packed, ill-defined vague outlines, active growth, larger and darker-staining nuclei, numerous mitoses, abundant blood vessels, minimal or no liquefaction necrosis in the 0 mCi and the control group (Fig. 3). The tumor cells were massively necrotic, homogeneous, red discoloration in the 0.6 mCi and 0.8 mCi groups. The normal cellular structure was basically invisible. The remaining cancer cells adjacent areas of necrosis were loosely arranged, karyolysis, no significant cytoplasmic staining, eosinophilic materia. Adjacent blood vessels were reduced, angiogenesis was not common (Fig. 3).

### Effect of ^125^I radiation on proliferation and apoptosis

**Fig. 4:**
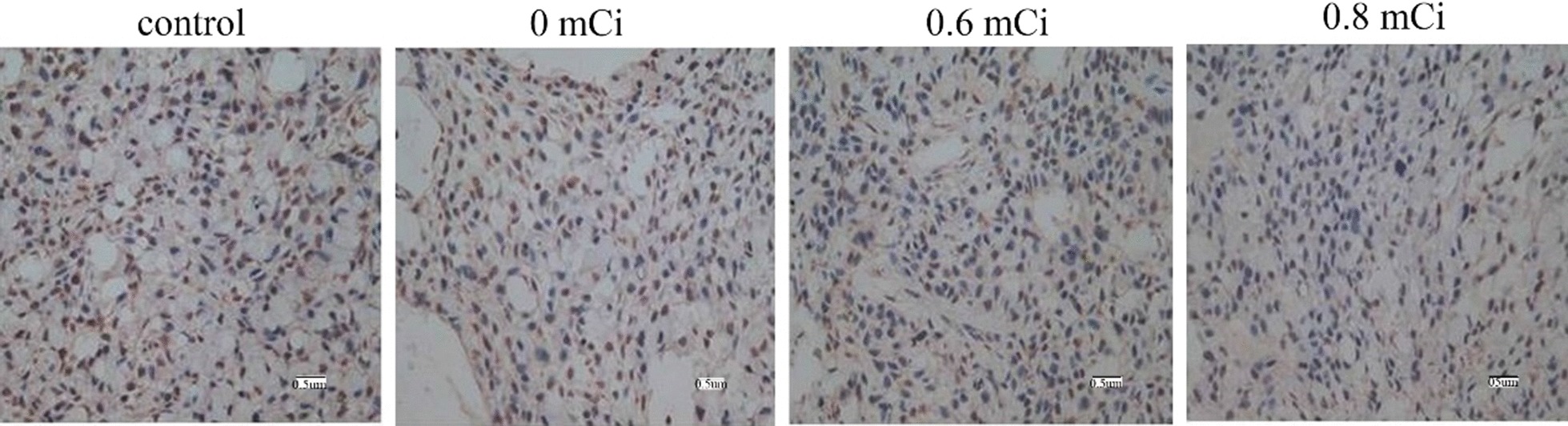
Tumors developing in nude mice stained with anti-Ki-67 monoclonal antibody (× 400)

Ki-67 was stained for nuclei, positive staining for cells was brown or tan in 0.6 and 0.8 mCi groups, mainly in the form of spots or lumps. These results indicated that low level and lighter brown cells in 0.6 and 0.8 mCi groups, while more positive staining for cells and dark brown in 0 and control groups (Fig. 4). We calculated the proliferation index of the tumor cells (Table 1). The proliferation index was substantially reduced in the 0.6 and 0.8 mCi groups than that in the control group (*P* < 0.05). However, no statistically significant difference was found between the 0.6 and 0.8 mCi groups (*P* > 0.05). The comparison between 0 mCi and control group was also no difference (*P* > 0.05). Here we show that 125I particles brachytherapy remarkably inhibits tumor cell proliferation.

**Table 1:** Proliferative index and apoptosis index expression in tumors ($\overline{x}$ ± s)%

Group	n	Proliferative index (%)	Apoptosis index (%)
Control group	10	71.00 ± 10.00	27.00 ± 4.69
0 mCi group	10	63.20 ± 6.22	35.50 ± 3.42
0.6 mCi group	10	46.20 ± 8.35*	50.00 ± 2.58*
0.8 mCi group	10	38.60 ± 6.03*	62.33 ± 4.51*
*F* value		31.853	45.34
*P* value		< 0.001	< 0.001

Compared with the control group, *P < 0.05

**Fig. 5:**
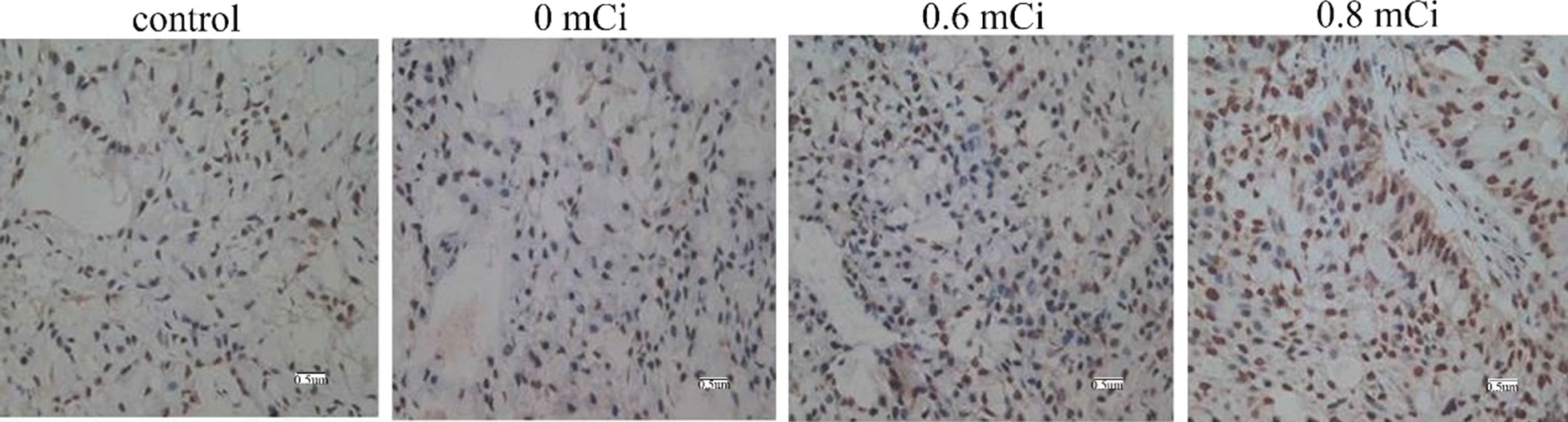
Sections of tumors developing in nude mice stained with TUNEL (× 400)

Under a light microscope, nuclear staining of apoptotic cells was in yellow–brown by TUNEL. The number of apoptotic cells in 0.8 mCi group and 0.6 mCi group were significantly higher than those in 0 and control group (Fig. 5). Compared with the control group, the 0.6 and 0.8 mCi groups had significantly higher apoptotic index (*P* < 0.05). The differences were not statistically significant between 0 mCi group and controls (*P* > 0.05). A comparison of the 0.6 mCi group and 0.8 mCi group showed no difference (*P* > 0.05). We examined the activity of caspase-3 and caspase-8 (Table 2). The activity of caspase-3 and caspase-8 in 0.6 mCi and 0.8 mCi groups was significantly higher than that in control group (*P* < 0.05). There was no significant difference between groups of 0 mCi and control (*P* > 0.05). The difference between 0.6 mCi and 0.8 mCi groups was not significant (*P* > 0.05). These results suggest that ^125^I implanted radiotherapy can significantly accelerate tumor cell apoptosis.

**Table 2:** Expression of caspase-3 and caspase-8 activities in tumors in each group ($\overline{x}$ ± s)%

Group	n	Caspase-3	Caspase-8
Control group	10	0.23 ± 0.03	0.32 ± 0.03
0 mCi group	10	0.21 ± 0.23	0.34 ± 0.12
0.6 mCi group	10	0.67 ± 0.12*	0.83 ± 0.49*
0.8 mCi group	10	0.70 ± 0.12*	1.15 ± 0.17*
*F* value		621.383	11.57
*P* value		< 0.001	< 0.001

Compared with the control group, *P < 0.05

### Expression of P21, caspase-9, survivin, livin proteins

**Fig. 6:**
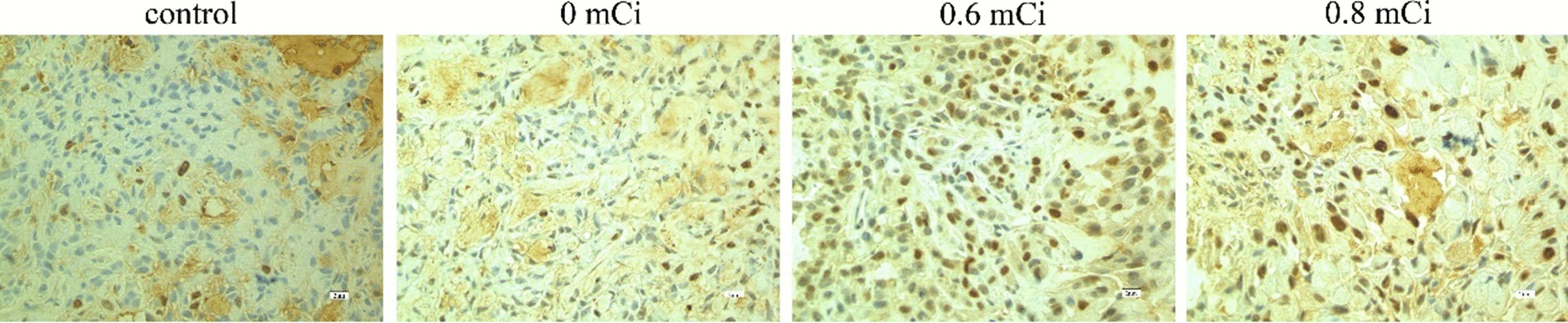
Immunocytochemistry of P21 protein in nude mice xenografts (S–P method × 400)

**Fig. 7:**
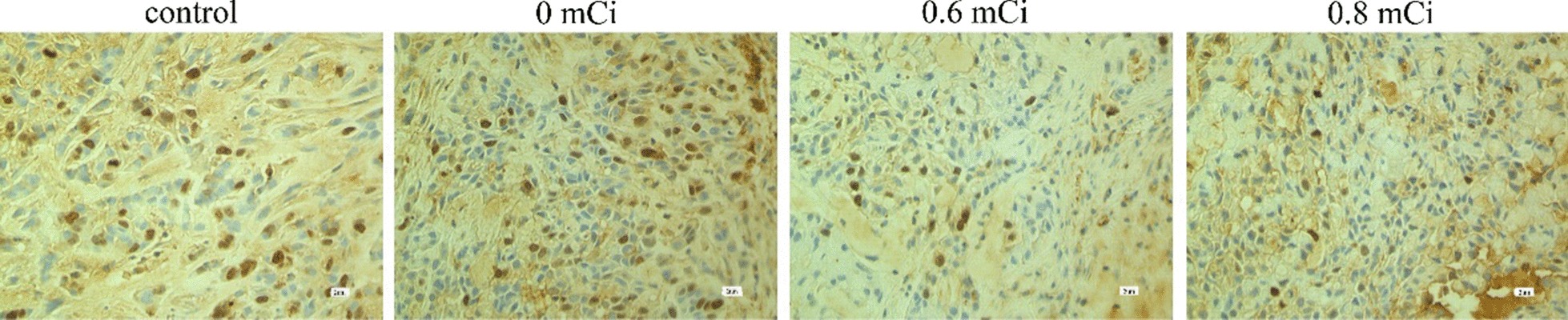
Immunocytochemistry of survivin protein in nude mice xenografts (S–P method × 400)

**Fig. 8:**
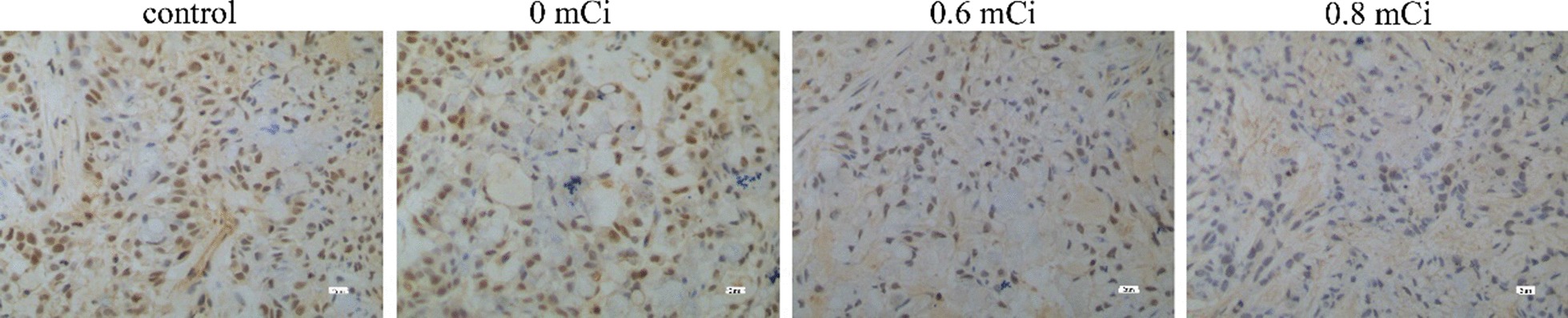
Immunocytochemistry of livin protein in nude mice xenografts (S–P method × 400)

**Fig. 9:**
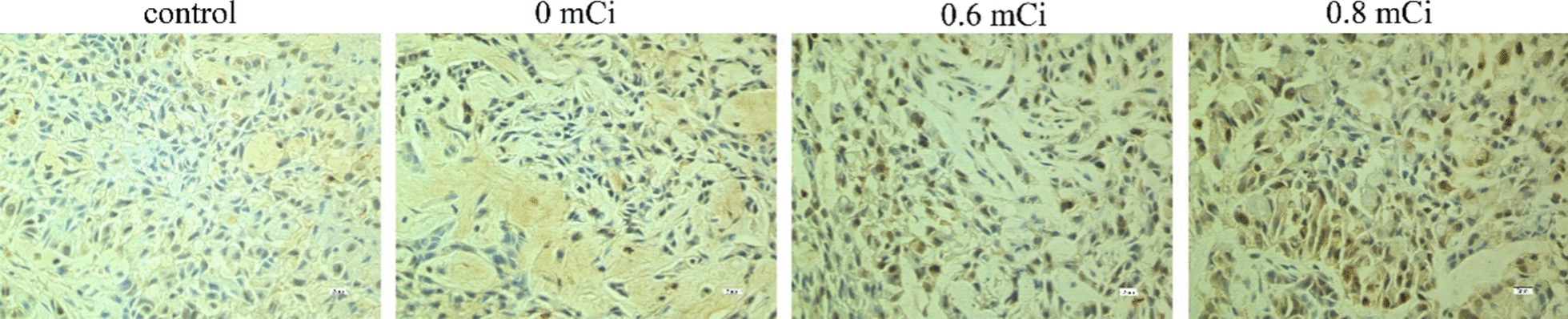
Immunocytochemistry of caspase-9 protein in nude mice xenografts (S–P method × 400)

After treatment in the animals of all four groups, we determined the P21, survivin, livin and caspase-9 protein expression in A549 xenograft tumors by immunohistochemistry (Figs. 6, 7, 8, 9). The positive rate P21, survivin, livin and caspase-9 protein expression in four groups was calculated (Table 3). In groups 0.6 mCi and 0.8 mCi, the positivity rate of P21 and caspase-9 protein expression was significantly higher compared to the control group (*P* < 0.05), while survivin and livin markedly lower than in control group (*P* < 0.05). There was no statistically significant difference in the expression of P21, caspase-9, survivin and livin between the 0 mCi group and the controls (*P* > 0.05). The four proteins were not different between the groups 0.6 mCi and 0.8 mCi (*P* > 0.05).

**Table 3:** Protein expression in tumors ($\overline{x}$ ± s)%

Group	n	Protein expression			
		P21 (%)	Caspase-9 (%)	Survivin (%)	Linvin (%)
Control group	10	16.7	33.3	77.8	83.3
0 mCi group	10	22.2	38.9	66.7	77.8
0.6 mCi group	10	66.7*	77.8*	27.8*	33.3*
0.8 mCi group	10	88.9*	88.7*	6*	16.7*

Compared with the control group, *P < 0.05

## Discussion

Although significant progress has been made in surgery, radiotherapy and chemotherapy in recent years, the 5-year survival rate is still very poor for advanced lung cancer. The role of radiation therapy in the treatment, plays an important role of advanced stage non-small-cell lung cancer. Interstitial brachytherapy with radioactive seeds has a history spanning more than 100 years [35]. The anti-tumor effect of 125I seed has been studied in recent years, as well as the mechanism of 125I seed for induction of apoptosis and cell cycle inhibition [19, 20, 27–29, 33, 36], DNA hypomethylation [19, 30] and anti-angiogenesis [27, 37, 38]. The most extensively studied mechanism is apoptosis [39, 40]. More recently, studies have shown that apoptosis and inhibiting proliferation may play an essential role in the treatment effects of ^125^I, but their mechanism of action has not been determined completely. Blocking in apoptosis may confer a survival advantage on malignant cells harboring genetic mutations and thus promote cancer progression [41]. It is likely that mitochondrial related autophagy disturbed mitochondria-dependent apoptotic pathway to delay apoptosis [42]. In radiation therapy, radiation can induce autophagy in normal and cancer cells [43–46].

Survivin is a well-known protein that belongs to the family of the inhibitor of apoptosis proteins (IAP) family which can regulate of tumor cell division and apoptosis inhibition. It is encoded by the BIRC5 gene located on the chromosome 17q25 [47]. Livin has been identified as a new member of the IAP family which was first identified in melanoma samples and was named melanoma inhibitor of apoptosis [48]. Livin, a member of the inhibitors of apoptosis proteins, is overexpressed in tumor tissues and is detected at substantially lower levels or not expressed at all in corresponding normal tissues. Its expression is considered a poor prognostic marker [41, 49]. P21 is one of the most important negative regulatory factors in the cell cycle and also plays a very important role in the process of apoptosis and cell proliferation [50–52]. P21 mainly regulate the activity of intracellular CDK (cyclin dependent kinase) leaving the cells in the G1 or G2 phase [50–52], through the regulation of tumor suppressor gene p53, but also by p53-dependent manner by other factors induced by the production. Ki67 has been described as a reliable indicator in the rate of cell proliferation, and it is a proliferation marker expression in cells expression throughout all stages except G0 phase [48].

The central event in apoptosis is the proteolytic activation of a class of cysteine aspartyl-specific proteases(Caspase) family [41, 48, 53–58]. Caspases are known to act as important mediators of apoptosis and contribute to the overall apoptotic morphology by the cleavage of various cellular substrates [47, 52, 57]. Caspase-3 is an important regulator of apoptosis and key enzyme in the Caspase family, and most factors initiate apoptosis through caspase -3-mediated pathway [38, 47]. Caspase-3 is the main performer of the apoptotic procedure, activating DNA fragments, leading to DNA degradation, resulting in nuclear fragmentation and inducing apoptosis by cascade reactions [41]. This gene encodes a protein that belongs to a highly conserved family of cysteinyl aspartate-specific proteases that function as essential regulators of programmed cell death through apoptosis. The increase of caspase-3 expression may induce the mitochondria-dependent apoptotic pathway with the possible mitochondria-independent pathway for the caspase-8 activation for that caspase-8-mediated apoptosis induced by oxidative stress is independent of the intrinsic pathway and dependent on cathepsins [41, 48, 53–58]. The intrinsic pathway of apoptosis is associated with the activation of caspase-9, which cleaves and activates caspase-3 [52–57].

We investigated the mechanism of 125I seed in treating lung cancers by establishing and using an animal transplant tumor model. Our results demonstrated that a higher absorbed dose of 125I induced a higher percentage of apoptosis. The results confirmed that 125I treatment induced tumor cell apoptosis, with decreasing P21, Ki-67, survivin, livin level expression, increased Caspase-9 expression and elevated caspase-3 activation. Implantation of 125I particles resulted in a decrease of Ki-67 expression in the tumor, thereby inhibiting cell proliferation. P21, survivin and livin all can affect apoptosis by inhibiting caspase-3 at the same time. Livin is recruited to death receptor signaling complexes, where it inhibits activation of caspases responsible for apoptosis and protects cells from diverse pro-apoptotic stimuli [41, 48]. Livin interacts with downstream caspases, such as caspase-3 and caspase-9, leading to their inactivation and degradation [48]. It has been suggested that antisense oligonucleotide of livin could promote cancer cell apoptosis by increasing the caspase-3-mediated apoptosis pathway [41, 48].

Moreover, p21 shields the cancer cells from death induced by DNA-damaging agents, and altered p21 expression increases sensitivity to treatment in vivo [50, 52]. P21 is an apoptosis regulatory factor, which is due to inhibition of the activity of procaspase-3 [51]. The antiapoptotic effect of survivin is connected with the activation of caspase-3. Survivin can directly inhibit the activity of caspase-3 in downstream of apoptotic pathway and indirectly inhibit the activation of caspase-3 by caspase-9 [53–58]. The tumor xenografts after 125I radiation treatment, the expression of survivin and livin protein decreased, while the expression of P21 increased. The effect of all three on caspase-3 was weakened at the same time, thereby inhibited the activity and function of caspase-3 and promoted the apoptosis of A549 cells.

Of course, our research has some limitations. First, all lung malignancies include squamous cell, adenocarcinoma, large cell, and small cell lung cancer. Lung cancer includes many subtypes such as adenocarcinoma, squamous cell carcinoma, and small cell carcinoma, and only one type of adenocarcinoma was selected from Lung cancer and the sample size of each group was small. A study has shown that squamous lung tumor is sensitive to 125I seed irradiation, and the treatment effect is significant [59]. Small cell lung cancer is also effective for 125I seed irradiation [59], but here is little information on other pathologic lung tumor types of 125I seed irradiation except NSCLC. Second, the inhibitory effect of 125I on NSCLC is known entity, and we have only initially studied 125I to inhibit lung cancer through apoptosis and the mechanism of interaction is unclear. Third, there are multiple human lung cancer cell lines and further study is needed because our study only included A549 human lung tumor xenografts.

In conclusion, our study successfully establishes the mouse lung adenocarcinoma model and provides a beneficial exploration of radiobiology of continuous different dose 125I seed irradiation in the treatment of lung adenocarcinoma. 125I seed implantation effectively inhibited tumor growth and reduced tumor volume, thus reducing tumor volume and improving the quality of animal survival. 125I irradiation inhibited the proliferation and induced cell apoptosis is the key mechanisms underlying the therapeutic effect of 125I seed implantation [39, 40]. In the tumor microenvironment, 125I irradiation can inhibit cell proliferation by reducing the levels of Ki-67, as well as inducing apoptosis by increasing the level of P21 and reducing the levels of survivin and livin. Although the mechanism of 125I particles in the treatment of tumors is not completely elucidated and many problems remain to be addressed, with further development of fundamental research, the application of 125I seed implantation in clinical practice will continue to be improved, in order to be better applied in clinical practice.

## Data Availability

The datasets used and/or analyzed during the current study are available from the corresponding author on reasonable request.

## Abbreviations

Ki-67: Nuclcar- associated antigen
P21: Protein 21
Caspse: Cysteine aspartyl-specific proteases
NSCLC: Non-small cell lung cancer
SCLC: Small cell lung cancer
H&E: Hematoxylin and eosin
TUNEL: Terminal deoxynucleotidyl transferase-mediated biotinylated UTP nick end labeling
DAB: Diaminobenzidine
IHC: Immunohistochemistry
IAP: Inhibitor of apoptosis proteins
