# Correction to: ^125^I seeds irradiation inhibits tumor growth and induces apoptosis by Ki-67, P21, survivin, livin and caspase-9 expression in lung carcinoma xenografts

**DOI:** 10.1186/s13014-021-01926-y

**Published:** 2021-11-06

**Authors:** Qing Jin, Cunzhi Lin, Xinhong Zhu, Yiwei Cao, Caihong Guo, Lijun Wang

**Affiliations:** 1 Department of Respiratory and Critical Care Medicine, The Afliated Hospital of Qingdao University, Qingdao, 266003 Shandong China; 2 Department of Critical Care Medicine, The 903Th Hospital of PLA Joint Logistics Support Force, Hangzhou, 310013 Zhejiang China; 3 Department of Internal Medicine, Qingdao Municipal Hospital, Qingdao, 266071 Shandong China

## Correction to: Radiat Oncol (2020)15:238 https://doi.org/10.1186/s13014-020-01682-5

After publication of this article [1], the authors reported that the captions for figures 3–5 had to be corrected to acknowledge the original source. The Acknowledgements section has been corrected too. The correction does not have any effect on the results or conclusions of the paper.

The original article [1] has been updated.
